# Invasive aspergillosis in autoimmune inflammatory rheumatic diseases: epidemiology, risk factors, diagnosis, management and challenges

**DOI:** 10.1080/07853890.2026.2685285

**Published:** 2026-06-25

**Authors:** Peixuan Liang, Xinyue Zhang, Shaozhe Cai, Ziwei Hu, Lingli Dong

**Affiliations:** ^a^ Department of Rheumatology and Immunology, Tongji Hospital of Tongji Medical College of Huazhong University of Science and Technology, Wuhan, China; ^b^ Department of Rheumatology and Immunology, Tongji Hospital, Tongji Medical College and State Key Laboratory for Diagnosis and Treatment of Severe Zoonotic Infectious Disease, Huazhong University of Science and Technology, Wuhan, Hubei, China

**Keywords:** *Aspergillus*, invasive aspergillosis, fungal infection, autoimmune inflammatory rheumatic diseases, immunomodulation

## Abstract

**Background:**

Invasive aspergillosis (IA) is a life-threatening opportunistic fungal infection caused by *Aspergillus* species. In recent years, IA appears to have become more frequently reported among patients with autoimmune inflammatory rheumatic diseases (AIIRD), likely reflecting the broader use of immunosuppressive therapies, with incidence in high-risk AIIRD subgroups reported to reach approximately 6.7% in selected cohorts.

**Objective:**

This review aims to summarize the current evidence on the epidemiology, susceptibility mechanisms, risk factors, clinical presentation, diagnosis, and management of IA in AIIRD, and to outline the clinical practical challenges in this population.

**Methods:**

This narrative review was informed by a structured literature search of PubMed, Embase, Web of Science, and Google Scholar for studies on IA in AIIRD published up to August 2025.

**Results:**

IA in AIIRD patients generally appears to arise from multiple interacting factors, including compromised host immunity, immunosuppressive therapy, the underlying rheumatic disease itself, comorbidities, and environmental exposures. *Aspergillus* infection and the resulting anti-*Aspergillus* immunity may also induce or exacerbate autoimmune inflammation. Invasive pulmonary aspergillosis is the most commonly reported manifestation, typically presenting with nonspecific respiratory symptoms, and disseminated infection tends to occur in the setting of profound immunosuppression. Early, integrated microbiologic testing (e.g. serum or bronchoalveolar lavage galactomannan, culture, polymerase chain reaction, and next-generation sequencing) together with serial imaging examination may facilitate earlier detection and guides care. Although robust AIIRD-specific evidence remains limited, current practice generally favour a multidisciplinary, individualized approach incorporating timely antifungal therapy and careful modulation of immunosuppression. Reported mortality remains high, ranging from 25% to 85% across AIIRD cohorts, particularly when diagnosis and treatment are delayed.

**Conclusions:**

IA is a serious and likely under-recognized infection in AIIRD patients. Multiple determinants appear to increase infection risk, and symptoms and imaging manifestations can mimic rheumatic disease activity, potentially contributing to diagnostic delay. Current epidemiological and clinical data on AIIRD-IA remain limited, and further studies are needed to refine risk stratification, establish diagnostic criteria tailored to AIIRD patients, and inform more evidence-based management strategies.

## KEY MESSAGES

1. Invasive aspergillosis (IA) appears to be an under-recognized and increasingly reported opportunistic infection in patients with autoimmune inflammatory rheumatic diseases (AIIRD), likely arising from multiple interacting factors, including impaired host immunity, immunosuppressive treatments, the underlying rheumatic disease, comorbidities, and environmental exposure.
2. Diagnosis of IA in AIIRD is challenging, as clinical and radiological features often overlap with rheumatic disease manifestations; timely use of advanced diagnostics (e.g., BALF galactomannan, polymerase chain reaction, and metagenomic sequencing) may be crucial for early recognition and improved outcomes.
3. Although robust AIIRD-specific evidence is currently lacking, management generally requires a careful balance between effective antifungal therapy and appropriate adjustment of immunosuppressive regimens; a multidisciplinary, individualized approach is widely advocated to address both infection control and rheumatic disease activity.

## Introduction

Invasive aspergillosis (IA) is among the most common causes of invasive fungal disease and is characterized by rapid progression and a poor prognosis [1]. It typically occurs in patients with severely compromised immunity or other debilitating conditions, with reported mortality rates as high as 85% [2]. Although IA has been widely documented in patients with haematological diseases, solid organ tumours, organ or stem-cell transplants, and in critically ill intensive care unit (ICU) population [3], its occurrence in patients with autoimmune inflammatory rheumatic diseases (AIIRD) has received comparatively less attention. In AIIRD, IA mostly manifested as invasive pulmonary aspergillosis (IPA) with non-specific clinical symptoms and atypical imaging findings that frequently overlap with the underlying rheumatic disease, which may contribute to diagnostic delays or post‑mortem identification [4,5]. Currently, there are no established diagnostic or treatment guidelines specific to IA in AIIRD, and existing knowledge is derived mainly from case reports and retrospective studies.

This narrative review synthesizes published evidence on IA in AIIRD through a structured literature search. We did not conduct formal systematic review methodology with quality scoring or meta-analysis, as the available evidence comprises primarily case reports and small retrospective series not amenable to such approaches. We searched PubMed, Embase, Web of Science and Google Scholar for studies published up to August 2025. Search terms combined “invasive aspergillosis” with AIIRD-related terms (e.g. “autoimmune inflammatory rheumatic diseases”, “connective tissue diseases”, “rheumatoid arthritis”, “systemic lupus erythematosus”, “vasculitis”, or “idiopathic inflammatory myopathy”, etc.). The selection process involved an initial screening of titles and abstracts, followed by a comprehensive review of the full-text articles. We include case reports, case series, observational studies, and relevant systemic reviews that provided epidemiological or clinical data on IA in AIIRD. Exclusion criteria included non-English publications, duplicate publications, and articles lacking clear IA confirmation. IA was assessed based on the consensus definitions of the European Organization for Research and Treatment of Cancer/Invasive Fungal Infections Cooperative Group and the National Institute of Allergy and Infectious Diseases Mycoses Study Group (EORTC/MSG; 2008) [6], and the revision and update of the European Organization for Research and Treatment of Cancer and the Mycoses Study Group Education and Research Consortium (EORTC/MSGERC; 2020) [7]. Cases were classified as proven, probable, or possible on the basis of histopathological evidence, host factors, compatible clinical/radiologic features, and mycological findings. To minimize misclassification, only proven or probable IA were included. Possible IA, colonization, and non-invasive forms were excluded. We extracted data including study design, patient characteristics (e.g. AIIRD type, immunosuppressive therapy, risk factors of infection or poor prognosis, etc.), clinical manifestations, and outcomes. Two authors performed eligibility screening of identified records and data extraction from the included studies, and any discrepancies were resolved through discussion or consultation with a third reviewer. The principal findings are presented as an integrative narrative synthesis of the available literature, with the studies underpinning this synthesis summarized in an evidence-summary table (Supplementary Table S1).

Based on the retrieved literature and extracted data, we summarize the epidemiology, pathogenesis and susceptibility factors, clinical manifestations, diagnosis, and treatment strategies for IA in AIIRD. We also discuss the diagnostic and therapeutic challenges in this population, limitations of existing criteria, and future directions in this field.

## 1. Epidemiology

In recent years, with the widespread use of immunosuppressive therapy, an increasing number of cases of IA have been reported in patients with AIIRD [8,9]. *Aspergillus fumigatus* is the most frequently identified causative species, followed by *Aspergillus flavus* and *Aspergillus niger* [10]. The reported incidence of IA in AIIRD patients ranges from 0.1% to 6.7%, generally higher than that in the general population [9,11–15]. However, this wide range should be interpreted with considerable caution. The available estimates are derived almost exclusively from retrospective studies that differ in patient selection, underlying disease severity, immunosuppressive exposure, referral patterns, and the diagnostic methodology applied to confirm IA. A French multicenter cohort (RESSIF registry, 2012–2018) identified 549 autoimmune disease patients with invasive fungal diseases, among which 84 (15.5%) were IA (third most common after *Pneumocystis* pneumonia and candidemia), with rheumatoid arthritis (RA) and anti-neutrophil cytoplasmic antibody (ANCA)-associated vasculitis (AAV) being the most common underlying diseases [9]. In a single-center retrospective study in China, 32 cases of invasive fungal infections were diagnosed among 6,911 patients with connective tissue diseases, with *Aspergillus* species accounting for 81.3%, being the most common pathogen. Among these, AAV showed a relatively higher incidence (1.5%) [16]. Across the available studies, IA has most often been reported in systemic lupus erythematosus (SLE), AAV, and RA [9,12,16,17], followed by idiopathic inflammatory myopathy (IIM) [11]. Other AIIRD, such as Sjögren’s syndrome (SS), systemic sclerosis (SSc), and spondyloarthropathies (SpA), appear to have a lower reported occurrence, with only isolated case reports currently available [18–20]. Variations in IA prevalence among AIIRD subgroups may be attributable to differences in the prevalence of the underlying diseases, disease activity and intensity of immunosuppressive agents; however, whether a true disease-specific susceptibility gradient exists remains uncertain and warrants further investigation in dedicated cohort studies.

Once IA develops, the clinical prognosis appears to be poor. Reported mortality rates of IA in AIIRD patients range from 25% to 85% [2,11–13], which may depend on the study population, the degree of immunosuppression, diagnostic timing, and antifungal management. In the RESSIF cohort, 30-day and 90-day mortality rates were 28.4% and 39.2%, respectively [9]. Encouragingly, recent improvements in diagnosis and management have contributed to declining overall mortality trend in IA patients [21].

To date, the epidemiology of IA in AIIRD patients remains incompletely defined, as the existing evidence is mainly derived from retrospective studies or small case series. Large prospective studies with standardized case definitions and more comparable patient populations are required to better define the disease burden and clinical features in this population. A concise, disease‑specific summary of epidemiology, clinical presentation, treatment strategies, infection risk factors, and poor prognostic factors across the major AIIRD is presented in Table 1.

**Table 1.** The epidemiology, clinical presentation, treatment strategy, potential infection factors and poor prognostic factors of invasive aspergillosis (IA) in autoimmune inflammatory rheumatic diseases (AIIRD).

Disease	Epidemiology	Clinical presentation	Treatment Strategy	Infection Factors	Prognostic Factors	References
Systemic lupus erythematosus	Prevalence: 0.11% − 1.4% Mortality: 35.7% − 66.7%	Organ involvement: predominantly pulmonary, and central nervous system involvement (e.g. the cerebellum and meninges) has also be documented. Clinical features: fever, cough, sputum, hemoptysis, dyspnea, and necrotic skin lesions. Clinical presentation can be insidious and hard to distinguish from lupus or coexisting infections. Imaging findings: nodules, consolidation, ground-glass opacities, and cavitation are common.	Voriconazole is first-line therapy in most cases. Immunosuppressive medications are generally suspended or reduced during infection treatment. Supportive care (e.g. ventilatory support for respiratory failure, lesion resection if needed) and therapy for concomitant infections are often required in parallel.	Use of mycophenolate mofetil^b^ Use of cyclosporine^b^ Use of (intravenous) glucocorticoid^a^ High accumulated dose of glucocorticoid^b^ Current glucocorticoid dose^a^ Granulocytopenia^b^ Lymphopenia^a^ Renal impairment^b^ Active lupus nephritis^b^ High disease activity / High SLEDAI-2K score^a^ Short disease duration^b^	High daily steroid dose (e.g. prednisone dose > 20 mg/d)^b^ Recent pulse steroid therapy^b^ Use of azathioprine^b^ Use of rituximab^b^ Concurrent infections^b^ Cytomegalovirus viremia^b^ Leukopenia^b^ Neutropenia^b^ High anti-dsDNA antibodies^b^ High SLEDAI-2K score^b^	Su et al. [12] Kim et al. [15] Huang et al. [121] Katz et al. [182] Kunawathanakul et al. [103] Lao et al. [183] Silva et al. [184]
Rheumatoid arthritis	No precise epidemiological data. In a French multicenter series, rheumatoid arthritis was the most frequent autoimmune rheumatic disease associated with invasive aspergillosis (15/84, 18%).	Organ involvement: mainly pulmonary involvement; sinus or disseminated involvement is rare. Clinical features: patients with rheumatoid arthritis often have underlying pulmonary conditions such as interstitial lung disease or cavitary lesions which can serve as foci for *Aspergillus* infection.	Voriconazole (or isavuconazole) is the first-line antifungal treatment. Liposomal amphotericin B is used in some cases or as salvage therapy. All biologic therapies (especially TNF-α inhibitors) and potent immunosuppressants should be withheld. Surgical intervention may be considered if focal lesions persist despite therapy or to prevent hemoptysis.	Long-term tumor necrosis factor (TNF) inhibitors (e.g. adalimumab, infliximab, and etanercept)^b^	--	Galmiche et al. [9] Barbosa et al. [64]
Antineutrophil cytoplasmic antibody-associated vasculitis	Prevalence: 4.5% Mortality: 57.1%	Organ involvement: primarily lung. About 87% (40/46) of reported vasculitis-related IA cases had invasive pulmonary aspergillosis. Clinical feature: hemoptysis, pleuritic pain, and fever that can mimic a vasculitis flare. Imaging findings: consolidation, cavitary lesions, nodules, and haemorrhagic pulmonary bronchopneumonia.	First-line antifungal agents include voriconazole. Immunosuppressive therapy such as cyclophosphamide or rituximab should be temporarily halted or tapered.	Long-term glucocorticoid treatment ^c^ Recent high-dose glucocorticoid and cyclophosphamide therapy ^c^ Chronic pulmonary disease (e.g. lung cavities, tissue necrosis, micro-haemorrhages) ^c^	Older age^b^ Underlying chronic respiratory diseases or more severe lung injury^b^ Lower hemoglobin level^b^	Baliga et al. [13] Su et al. [14]
Anti-melanoma differentiation antigen 5 (MDA5) antibody-positive dermatomyositis	Prevalence: 6.7% Mortality: 25%	Clinical features: cough, dyspnea, fever and chest pain. Anti-melanoma differentiation antigen 5 antibody-positive dermatomyositis patients who develop invasive aspergillosis typically have coexisting interstitial lung disease. Imaging findings: pulmonary nodules (more frequently observed in infection group), ground-glass opacities, consolidation, halo sign, cavity, and pleural effusion.	Voriconazole-based antifungal therapy. Combination therapy (e.g. azole with caspofungin) depending on severity and organism sensitivity. Given that dermatomyositis and rapidly progressive interstitial lung disease can be lethal, appropriate anti-inflammatory treatment and respiratory support are necessary.	Increased bronchoalveolar lavage fluid galactomannan levels^b^	Rapidly progressive interstitial lung disease^b^ Lower lymphocyte counts^b^ Co-infection with *Pneumocystis jirovecii*^b^	Chen et al. [11]
Systemic sclerosis	(Only isolated case reports)	Clinical feature: atypical worsening of cough, dyspnea, and hemoptysis on a background of fibrotic lungs. Imaging findings: bilateral lung infiltrates with cavitation in areas of prior honeycombing.	Treatment is guided by general principles including antifungal therapy and immunomodulation.	Parenchymal fibrosis combined with immunosuppressive therapy ^c^	--	Shadrach et al. [18]
Ankylosing spondylitis	(Only isolated case reports)	Clinical feature: worsening dyspnea, productive cough, weight loss, and lethargy. Imaging findings: cavitary lesions, infiltrates, adjacent inflammatory change and patchy air space opacity.	Treatment is guided by general principles including antifungal therapy and immunomodulation.	Pulmonary cavities ^c^ Pulmonary apical fibrosis ^c^ Severe influenza pneumonia history ^c^ Tumor necrosis factor inhibitors (infliximab) ^c^	--	Attaway AH et al. [19] Kennedy MP et al. [20]
Connective tissue disease	Prevalence: 0.38% Mortality: 26.9% (In one center, in patients with connective tissue disease-associated interstitial lung disease, 35% developed invasive pulmonary aspergillosis, with 55.6% of infection patients dying.)	Organ involvement: lung and the sphenoid sinus. Clinical feature: fever, cough, sputum, dyspnea, and hemoptysis. CT scan findings: small nodules and cavitary lesions were more common. Halo sign and air crescent sign were not frequently reported.	Treatment is guided by general principles including antifungal therapy and immunomodulation. Prolonged treatment (≥12 weeks) may be required in patients with chronic lung disease. Granulocyte colony-stimulating factor or intravenous immunoglobulin support may be considered in the presence of severe neutropenia or hypogammaglobulinemia.	Lymphopenia^a^ Median-to-high dose of glucocorticoid^b^ Pulmonary involvement (e.g. interstitial lung disease, chronic obstructive pulmonary disease)^b^ Use of multiple immunosuppressants (e.g. cyclophosphamide, calcineurin inhibitors, azathioprine, mycophenolate)^b^	Decreased CD3^+^ CD4^+^ T cells^b^ Lymphopenia^b^ Leukopenia^b^ Kidney impairment^b^ Co-infection^b^	Lao et al. [16] Xiong et al. [57] Shi et al. [167]

Notes.

^a^ consistent findings across multiple cohorts or studies.

^b^ reported in a single cohort or small retrospective study; ^c^ based mainly on case series, case reports, expert opinion or extrapolation. These indicators reflect the consistency of the available evidence rather than a formal evidence-grading system.

Abbreviations: anti-dsDNA, anti-double-stranded DNA; IA, invasive aspergillosis; SLEDAI-2K, Systemic Lupus Erythematosus Disease Activity Index 2000; TNF-α, tumor necrosis factor-alpha.

## 2. Antifungal immunity, susceptibility mechanisms and risk factors

**Figure 1.**
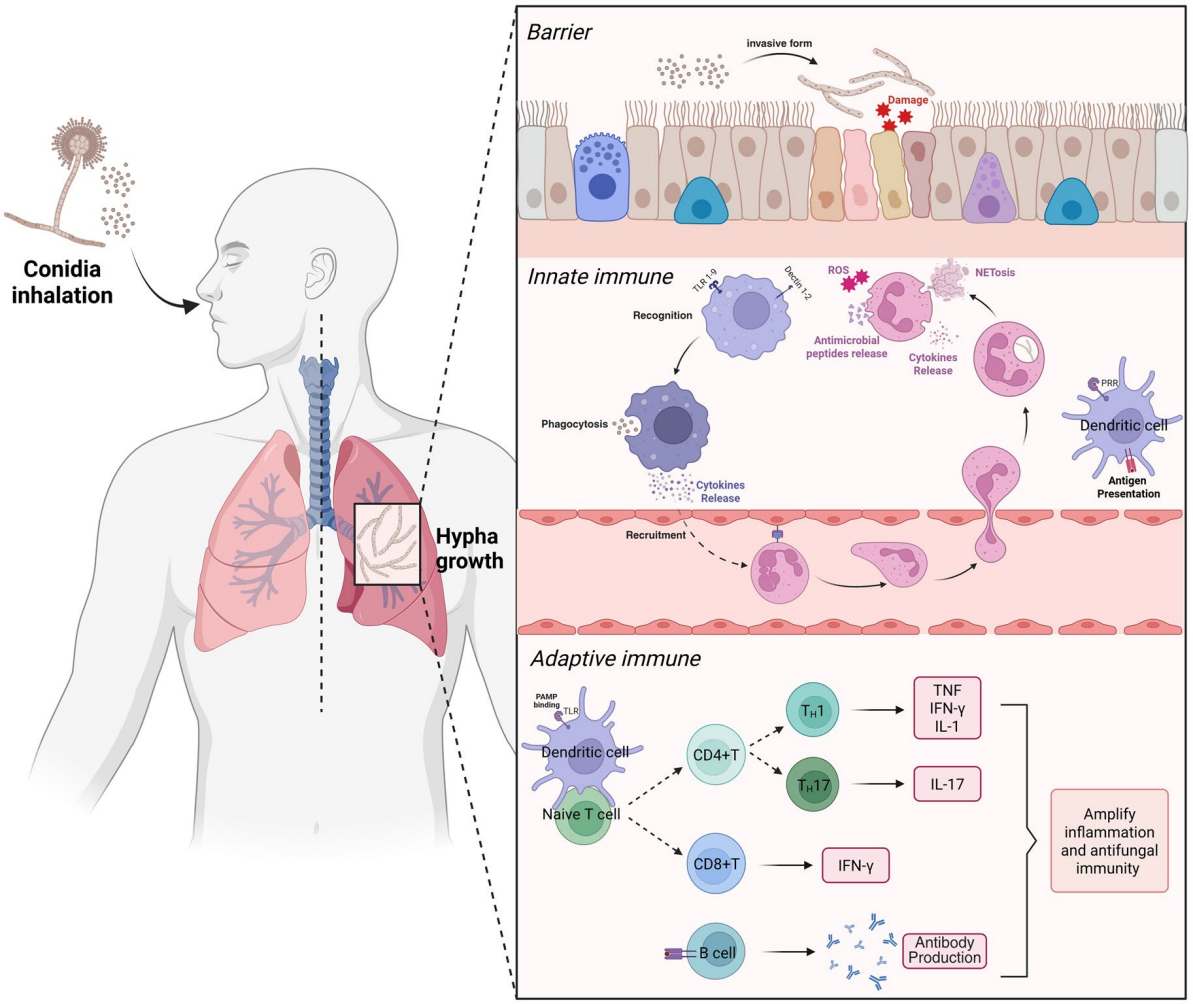
Three processes of defense against *Aspergillus* infection. **(a)** Barrier defense. Respiratory epithelial cells, mucus, and mucociliary clearance constitute the first line of defense against inhaled *Aspergillus* spores. Disruption of airway integrity may facilitate conidial germination, hyphal invasion, and tissue penetration. **(b)** Innate immune recognition. Fungal cell-wall components are recognized by pattern-recognition receptors, including C-type lectin receptors and Toll-like receptors, on epithelial and innate immune cells, leading to inflammatory signaling, cytokine and chemokine release, and recruitment and activation of phagocytes. **(c)** Adaptive immune response. Dendritic cells promote adaptive antifungal immunity through antigen presentation, with Th1/Th17 responses and B-cell-mediated humoral immunity contributing to fungal control. (Figure was made using BioRender.).

*Aspergillus* is a ubiquitous conditionally pathogenic filamentous fungus present in air and soil, and airborne conidia enter the host *via* the respiratory tract [22]. Whether infection occurs depends on the balance between host immunity and fungal virulence. Respiratory epithelial cells and mucociliary clearance constitute the first line of defence [23]. Conidia escaping this barrier are recognized by innate immune cells, such as alveolar macrophages, *via* pattern recognition receptors (PRRs), including Toll-like receptors (e.g. TLR-1, 2, 3, 4, 6), C-type lectin receptors (e.g. Dectin1, Dectin2), and NOD-like receptors [24,25]. This recognition activates NF-κB signaling and inflammasome pathways [26], prompting the release of pro-inflammatory cytokines (e.g. TNF, IL-1, IL-6, IL-23) and chemokines (e.g. CXCL1, CXCL2, CXCL9, CXCL10) [25], which promote phagocytosis and neutrophil recruitment [27]. Neutrophils eliminate fungal pathogens through phagocytosis, degranulation, reactive oxygen species (ROS) generation, antimicrobial peptide secretion, and the formation of neutrophil extracellular traps (NETs) [28,29], while amplifying inflammation through a positive-feedback loop that reinforces antifungal immunity [30,31]. Dendritic cells subsequently capture and present *Aspergillus* antigens to drive adaptive responses [24,32], with Th1, Th17, and CD8^+^T cells producing cytokines such as TNF, IFN-γ, IL-17, and GM-CSF that further activate phagocytes [33–36]. B cells contribute through antibody production, immune complex formation, and subsequent complement activation [1]. Figure 1 illustrates these key components of the antifungal immune response.

In AIIRD patients, IA generally appears to arise from the convergence of several interacting factors, including impaired host immunity, immunosuppressive treatments, underlying rheumatic conditions, comorbidities, and environmental exposure. Once host defence is compromised, inhaled conidia may transform into invasive hyphae capable of penetrating airways, lung tissue, and the vasculature, with potential systemic dissemination. Table 1 summarizes the previously reported risk factors for IA infection and associated mortality in AIIRD patients.

### 2.1. Host immune status

#### 2.1.1. Abnormalities of innate immunity

Genetic polymorphisms in innate immune receptors such as TLRs and Dectin have been linked to host susceptibility to *Aspergillus* infection [37], and Dectin-1 deficiency in murine models impairs neutrophil recruitment and fungal killing [38]. In AIIRD, monocytes from patients with SLE and RA have shown decreased expression and function of Dectin-1, potentially compromising the recognition and clearance of *Aspergillus* and thus elevating IA risk [39]. The immune dysregulation inherent to AIIRD, when combined with phagocyte dysfunction induced by immunosuppressive therapy, may further increase susceptibility to fungal infections [40]. Prolonged neutropenia (e.g. absolute neutrophil count < 0.5 × 10^9^/L for more than 10 days) is a well-established risk factor for IA, with the infection risk correlating positively with the severity and duration of neutropenia [41]. In AIIRD patients, neutropenia is commonly observed in those with drug-induced bone marrow suppression or immune-mediated haematological complications [42,43]. Moreover, dysfunctions in the complement system (such as deficiencies in complement receptors and complement factors in patients with SLE) may hinder pathogen clearance by weakening opsonization [44–46].

#### 2.1.2. Abnormalities of adaptive immune

Chronic autoimmune inflammation in AIIRD can lead to lymphocyte depletion and impaired effector function. In RA, sustained antigenic stimulation disturbs the Th1/Th2 balance and reduces T-cell receptor diversity, which may limit the pool of T cells capable of mounting effective antifungal responses [47,48]. Reductions in lymphocyte subsets (including CD4^+^ and CD8^+^ T cells) and impaired T-cell function have been reported in association with invasive fungal infection in AIIRD [11,49–51]. Aberrant B-cell differentiation in AIIRD patients can be manifested as a skewed memory B-cell repertoire and hyperactivation of the extrafollicular pathway, may further compromise the generation of high-affinity IgG responses to foreign antigens [52,53].

### 2.2. Immunotherapy-related factors

**Figure 2.**
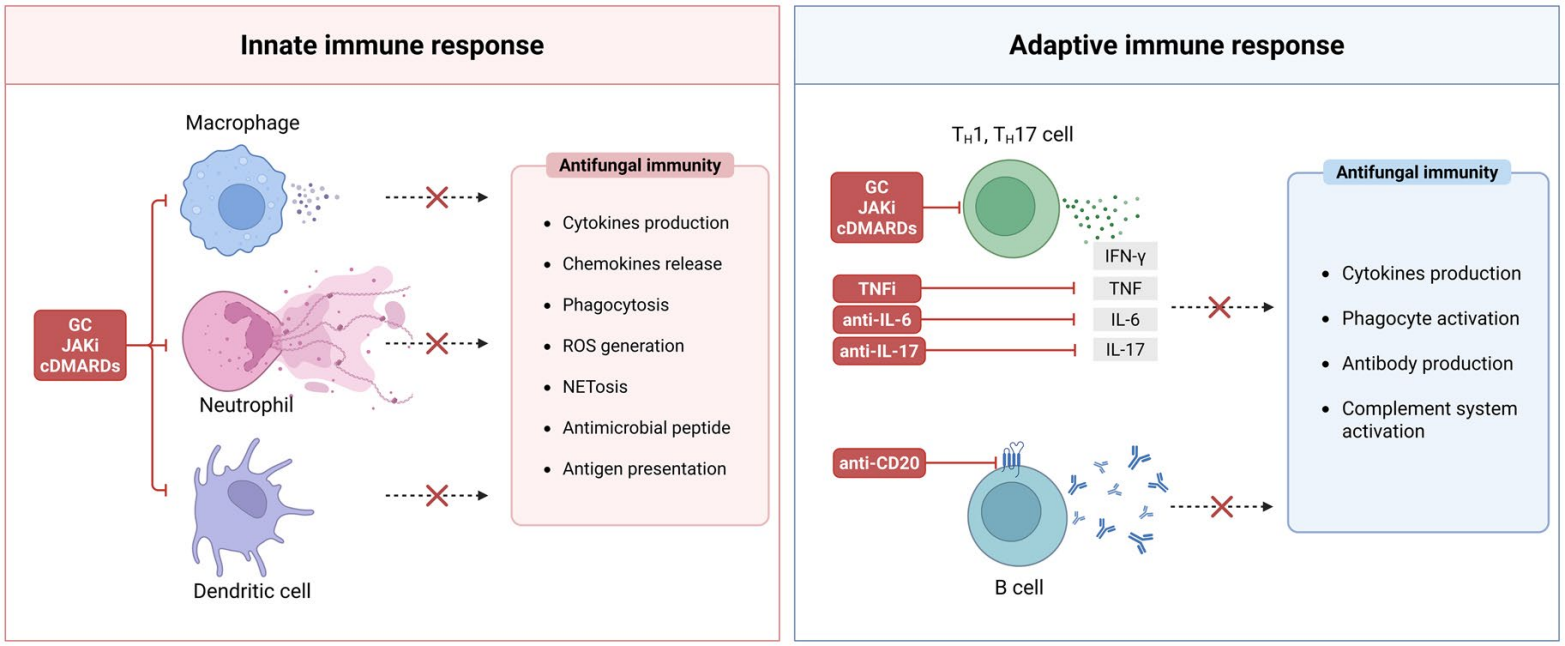
Effects of immunosuppressive agents on innate and adaptive antifungal immune responses in AIIRD patients. **Left panel (Innate immune response)**: GC, JAKi (e.g. tofacitinib, baricitinib, upadacitinib), and cDMARDs (e.g. CYC, CsA, FK506, MMF) suppress innate immune defenses by impairing macrophage, neutrophil, and dendritic cell functions. These drugs inhibit cytokine and chemokine production, phagocytosis, ROS generation, NETosis, antimicrobial peptide secretion, and antigen presentation, collectively weakening the host’s early antifungal immunity. **Right panel (Adaptive immune response)**: GC, JAKi, cDMARDs, TNF inhibitors (e.g. adalimumab, infliximab), anti-IL-6 biologics (e.g. tocilizumab) and anti-IL-17 biologics (e.g. secukinumab) reduce cytokine production (e.g. IFN-γ, TNF, IL-6, IL-17), impair phagocyte activation; anti-CD20 therapy (e.g. rituximab) diminish B-cell-mediated antibody responses, and limit complement system activation, thereby increasing susceptibility to fungal infections. (Figure was made using BioRender.).

Iatrogenic immunosuppression is among the principal risk factors for IA in AIIRD. Most patients who develop IA are on long-term glucocorticoids or other immunosuppressive drugs. The reported effects of these agents on antifungal immunity are summarized in Figure 2.

#### 2.2.1. Glucocorticoid (GC)

GC can compromise antifungal immunity by inhibiting macrophage and T-cell function and weakening the oxidative killing ability of neutrophils [54,55]. Several observational studies have linked GC exposure to IA in AIIRD. Intravenous GC administration was identified as a potential risk factor in one SLE cohort [12]; prednisone exposure ≥ 45 mg/day within the three months preceding infection has been linked with increased IA risk in SLE [8]; and high-dose prednisone (> 0.5 mg/kg/day) has likewise been reported as a contributing factor [56]. Although these findings suggest a probable association between higher GC exposure and IA risk, confounding by underlying conditions and by co-prescription of other immunosuppressants cannot be excluded. No validated GC threshold for IA risk stratification has been established in AIIRD, owing to heterogeneous exposure definitions and incomplete adjustment for confounders across studies. Clinicians should nonetheless weigh the potential benefits of intensive steroid therapy against infection risk on an individualized basis.

#### 2.2.2. Conventional disease-modifying antirheumatic drugs (cDMARDs)

In the French RESSIF network, more than half of AIIRD patients with invasive fungal infections received one or more immunosuppressants (IS) [9]. Agents such as mycophenolate mofetil (MMF), cyclophosphamide (CYC), and calcineurin inhibitors (including cyclosporine and tacrolimus) have been identified as potential risk factors for IA in observational studies and case reports [12,56,57]. In murine models, cyclophosphamide administration was associated with reduced pulmonary expression of Dectin-1 and increased fungal burden following *Aspergillus fumigatus* infection [58]. The cumulative effects of multiple immunosuppressive strategies should also be considered. Concomitant use of GC and IS or multiple IS may further increase the risk of opportunistic infections. A small number of observational studies have raised the possibility that hydroxychloroquine or methotrexate use may be connected with a lower incidence of invasive fungal infection [57,59]; however, this signal arises from heterogeneous populations and should be regarded as exploratory rather than as evidence of a protective effect that could inform prescribing decisions.

#### 2.2.3. Biologic and targeted synthetic disease-modifying antirheumatic drugs (b/tsDMARDs)

Tumour necrosis factor-alpha (TNF-α) plays an important role in antifungal host defense, including Th1 responses, macrophage activation, and leukocyte recruitment. Accordingly, TNF-α inhibition is plausible as a contributor to IA susceptibility [60]. A literature review identified 281 invasive fungal infections in patients receiving anti-TNF therapy for adult-onset Still’s disease, of which 64 (23%) were aspergillosis, with infliximab being the most frequently implicated agent [61]. Other studies similarly indicated that anti-TNF biologics accounted for over half of IA cases linked to biologic therapies [62], that infliximab and adalimumab accounted for the highest numbers of reported IA cases [63], and that the use of infliximab or etanercept was associated with IA occurrence in certain cohorts [64]. Collectively, these observational data suggest that anti-TNF therapy may represent an exposure associated with increased IA risk. Its independent role nevertheless remains uncertain due to confounding by concomitant therapies, underlying disease severity, and structural lung damage. In clinical practice, IA should remain in the differential diagnosis in AIIRD patients receiving TNF inhibitors, particularly when additional predisposing factors are present.

Rituximab (RTX) is a B-cell depleting agent that has been reported to reduce Th17-mediated antifungal responses and impair humoral immunity [65]. RTX treatment may induce late-onset neutropenia or hypogammaglobulinemia in a subset of patients, both of which are potentially linked to increased infection susceptibility [66]. These complications can develop weeks to months after the last infusion and are easily overlooked in the absence of scheduled post-infusion monitoring, and their contribution to IA risk in AIIRD remains incompletely characterized. Although IA sporadic cases in AIIRD patients using RTX have been reported, most occurred concurrently with additional immunosuppressive factors such as GC and combination immunosuppressants [67,68]. There are insufficient data to make conclusion on the role of RTX itself in the pathogenesis of IA. Moreover, other co-occurring susceptibility factors appear important. Underlying bronchial pathology such as bronchiectasis increases risks of *Aspergillus* colonization and invasion [69,70]. Concurrent viral respiratory infections such as severe influenza or coronavirus disease 2019 may further heighten the risk of aspergillosis in RTX induced immunosuppressed hosts by damaging airway defenses and provoking dysregulated inflammation [71–73].

Animal studies indicate that Janus kinase (JAK) 1/2 inhibitors may decrease neutrophil-mediated antifungal activity through suppression of JAK-STAT-dependent cytokine signalling [74], with downstream effects on ROS generation and NET formation [75,76], and may modulate dendritic cell-mediated Th17 response [77]. Clinical reports of IA in AIIRD patients receiving JAK inhibitors remain limited and are mainly confined to case-level descriptions [78–80]. Current evidence is insufficient to quantify the IA-specific risk of JAK inhibition, although a degree of clinical vigilance is appropriate given the regulatory warnings for serious infections associated with this class.

### 2.3. Underlying diseases and comorbidities

AIIRD inherently involves immune dysfunction that may predispose patients to IA. SLE has been linked to IA in several studies, with high SLEDAI scores, low complement levels, and the presence of anti-dsDNA antibodies reported as potential risk factors [8,15,56]. Patients with AAV may predispose to invasive fungal infections due to chronic structural lung changes, tissue necrosis, microhaemorrhage, and iron-rich environments [13,16]. In RA, an imbalance between protective and pathogenic Th17 cell subsets has been noted. Despite elevated baseline IL-17A levels in RA patients, their peripheral blood mononuclear cells (PBMCs) exhibit impaired fungus-induced IL-17A production, resulting in defective antifungal immune responses [81].

Pulmonary involvement is common in AIIRD [82]. For instance, RA, SSc, SS and IIM are frequently complicated by interstitial lung disease (ILD) [83–86]. Structural lung abnormalities such as pulmonary fibrosis and honeycombing impair mucociliary clearance and anatomical barriers [87]. Systemic vasculitis can cause cavitary lung lesions through chronic ischemic, creating a localized immunodeficient microenvironment (also known as “locus minoris resistentiae”) prone to fungal colonization [13]. Some patients with ankylosing spondylitis may develop fibrotic or cystic lesions in the upper lung lobes, which increase the risk of *Aspergillus* colonization [88]. The presence of other chronic pulmonary comorbidities in AIIRD patients also increase susceptibility to infection. Studies reported that patients with chronic obstructive pulmonary disease (COPD), bronchiectasis, pulmonary bullae, previous tuberculosis infection, cavity formation, or cystic fibrosis are prone to pulmonary *Aspergillus* infection. Patients with chronic obstructive pulmonary disease (COPD) have been identified as a high-risk group for non-neutropenic IA. Structural and functional abnormalities such as bronchiectasis, previous tuberculosis infection, cavity formation, and cystic fibrosis increase are prone to pulmonary *Aspergillus* infection [89–91]. Thus, an AIIRD patient with significant lung damage or chronic lung disease, especially those receiving intensive immunosuppression, should be monitored closely for possible IA.

Additional host factors associated with increased IA risk include advanced age, critical illness, malnutrition, diabetes mellitus, liver cirrhosis, chronic kidney disease, and the use of renal replacement therapy or mechanical ventilation [90,92–98]. Concurrent viral infections—including severe influenza, coronavirus disease 2019, and cytomegalovirus (CMV) infection—have also been linked with an increased risk of invasive pulmonary fungal disease [98–100]. This increased vulnerability may be attributed to viral infection-induced damage to the respiratory epithelial barrier and impaired phagocyte function [101,102].

In summary, AIIRD patients are exposed to multiple potential risk factors for IA related to host immunity, therapy and underlying diseases. Antifungal immunity may be compromised through abnormalities of innate immunity (notably macrophage and neutrophil function) and adaptive immunity (notably Th1 and Th17 responses), either as a consequence of the autoimmune process itself or of immunosuppressive therapy [3,22]. Based on current evidence, the most consistently reported risk signal involves GC exposure, particularly at high doses or over prolonged periods [8,12,56,103], with anti-TNF therapy [63,64] and combined use of potent immunosuppressants such as CYC, MMF and calcineurin inhibitors [12,56,57] may represent additional contributors. As most supporting evidence is retrospective and patients commonly received multiple concurrent immunosuppressants, the associations described above should be interpreted as exposure-related risk signals rather than evidence of independent causation by any single agent. Pre-existing structural lung disease constitutes an additional non-pharmacological risk factor that deserves attention [89–91]. In AIIRD, IA has been reported most frequently in SLE, RA and AAV, and particularly in patients with high disease activity [9,15–17]. For high-risk AIIRD populations, it is crucial to remain highly vigilant and conduct early fungal screening [104].

## 3. Anti-*Aspergillus* immunity induces and exacerbates autoimmunity and inflammation

The relationship between fungal infection and AIIRD appears to be bidirectional: immune dysregulation and immunosuppressive therapy predispose patients to fungal infections, whereas fungal pathogens may in turn exacerbate or initiate AIIRD through dysregulated immune activation [105]. Several mechanisms have been proposed to underlie this reciprocal interaction. Antifungal Th1/Th17 responses, although essential for host defense, may also amplify inflammation through cytokine release and oxidative stress [106–108]. In addition, *Aspergillus* and its mycotoxins may aggravate inflammation *via* inflammasome activation and ROS production [109–111]. For example, ochratoxin A has been shown to increase susceptibility to RA and intensify arthritis severity in collagen-induced arthritis models by enhancing macrophage activation and promoting Th1/Th17 responses [112], raising the possibility that mycotoxins may act as environmental exposure factors to initiate or exacerbate inflammatory arthritis. NETosis represents another double-edged mechanism: while it may help contain fungal spread, excessive NET formation can also promote inflammation, apoptosis, and tissue injury [1,28,113–115]. Fungal antigens may also break immune tolerance through molecular mimicry and epitope spreading, leading to abnormal activation of autoreactive B and T cells [105]. Thus, while antifungal immune activation is essential for pathogen clearance, dysregulated or prolonged inflammation may aggravate tissue injury, as exemplified by immune reconstitution inflammatory syndrome (IRIS) [116].

## 4. Diagnostic approaches

Currently, the most widely accepted diagnostic criteria for IA are the European Organization for Research on Treatment of Cancer/Mycoses Study Group Education and Research Consortium (EORTC-MSGERC) criteria [7], which encompass four parts: host (risk) factors, clinical features, histopathology, and microbiological examination. The diagnostic system is divided into three categories: proven, probable, and possible IA. The integrated diagnostic workflow described below, including the principal AIIRD-specific caveats, is summarized in Supplementary Figure S1 as a practical clinical reference.

### 4.1. Clinical manifestations and imaging features

**Figure 3.**
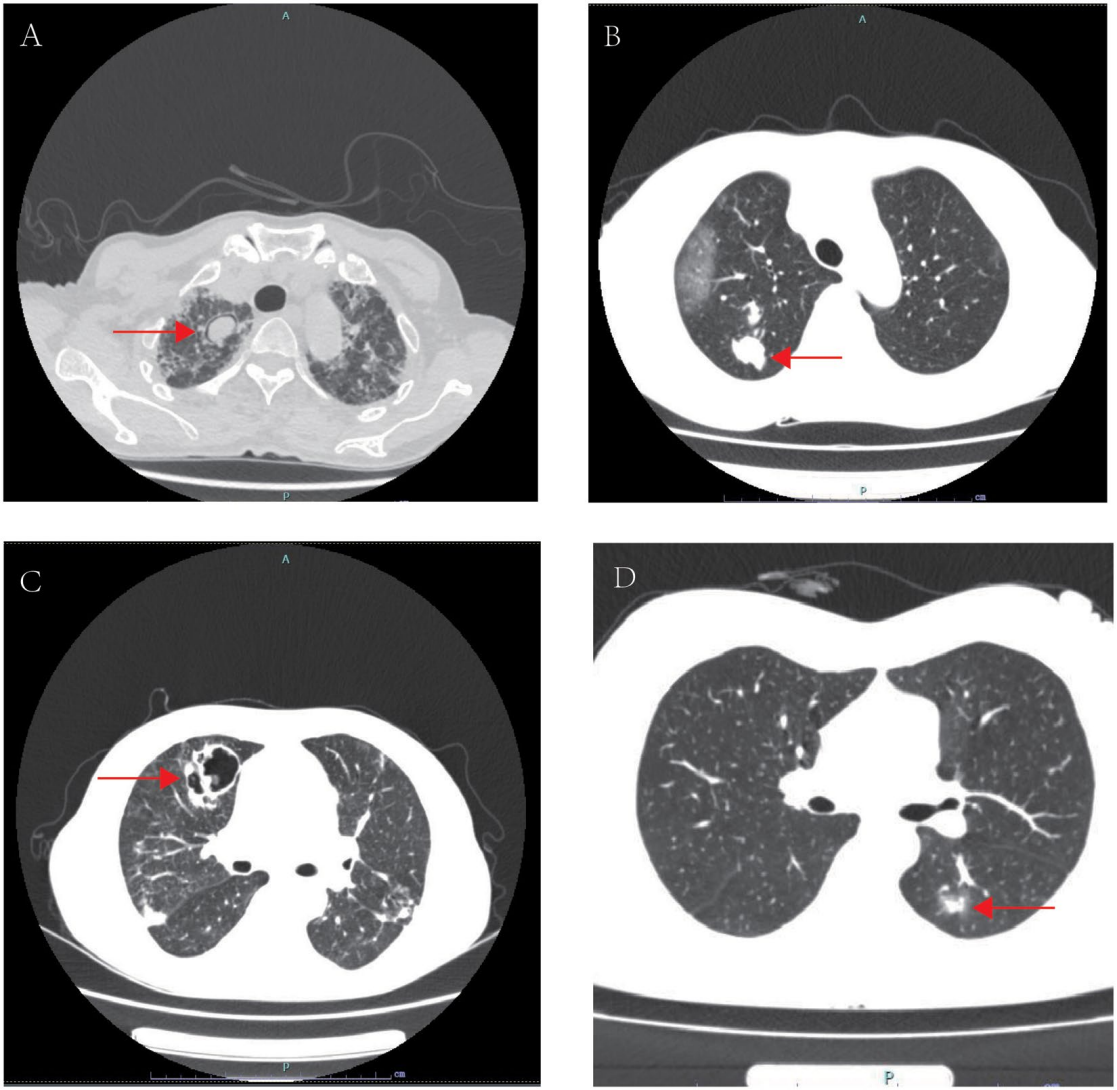
Representative imaging features of invasive pulmonary aspergillosis (IPA) in autoimmune inflammatory rheumatic disease (AIIRD) patients at diagnosis. **(A)** A patient with systemic lupus erythematosus showing an air‑crescent sign (red arrow). **(B)** A patient with rheumatoid arthritis showing a pulmonary nodule (red arrow). **(C)** A patient with antineutrophil cytoplasmic antibody–associated vasculitis showing a cavity (red arrow). **(D)** A patient with systemic lupus erythematosus showing a halo sign (red arrow). Note: Red arrows indicate the corresponding radiological signs in each panel.

Clinical and imaging features of IA often overlap with manifestations of active AIIRD. Common symptoms associated with IA include fever, cough, chest pain, dyspnoea, and hemoptysis, which are non-specific and can be mistaken for AIIRD flares [117]. Symptoms like fever may become atypical in some AIIRD patients due to GC or nonsteroidal anti-inflammatory drugs (NSAIDs) use. IA frequently coexists with other infections, such as viral or bacterial pneumonia, especially in AIIRD patients receiving long-term immunotherapy. Clinicians should suspect IPA in patients with progressively worsening respiratory status that does not respond to antibiotics or disease-directed therapy. Chest computed tomography (CT) is indicated in suspected instances of IPA, with high-resolution CT proving particularly informative [118]. Patients with neutropenia are more likely to exhibit characteristic halo signs and subsequent air crescent signs. However, the majority of AIIRD patients are non-neutropenic [119]. A lower sensitivity of halo sign and air-crescent sign (5–24%) in non-neutropenic immunosuppressed individuals has been reported [120]. Most AIIRD patients present with non-specific chest CT findings, such as nodules, ground-glass opacities, consolidation, patchy shadows, cavitations, wedge-shaped or lobar infiltrates, and bronchopneumonia patterns [5,11,119,121]. These findings can mimic bacterial pneumonia or AIIRD-associated lung involvement. Representative CT findings of IPA in AIIRD patients at diagnosis are illustrated in Figure 3, with key signs annotated.

Besides the lungs, IA can disseminate to virtually any organ, especially in patients with severe immunosuppression. Extrapulmonary disease most commonly involves the sinuses, brain, heart, bones, joints, eyes, and skin. Central nervous system involvement often presents with headaches, altered mental status, seizures, or neurological deficits [121], which are difficult to distinguish from neuropsychiatric symptoms of rheumatic diseases. Disseminated IA typically has a poor prognosis. Prompt imaging of any new symptoms (e.g. brain MRI for new neurological signs) is important to identify the full extent of disease. Imaging also helps in guiding biopsies and in monitoring response to therapy – for example, measuring the size and number of lesions over time.

### 4.2. Histopathology, microscopy and culture

Positive fungal evidence from sterile sites (such as lung tissue biopsies), or histopathological demonstration of *Aspergillus* hyphae with tissue damage are of confirmed diagnostic value [7]. Nonetheless, acquiring these samples can be challenging due to procedure invasiveness and risks in patients with thrombocytopenia or critical illness. Additionally, tissue culture has a low positivity rate, and both culture and histological staining are time-consuming, delaying early diagnosis [122]. Positive cultures obtained from non-sterile specimens, such as sputum or bronchoalveolar lavage fluid (BALF), should be cautiously interpreted, as results may represent contamination, colonization, or infection. Clinical significance is greater when positive findings are confirmed by two or more consecutive cultures [3].

### 4.3. Antigen testing

The main biomarkers for diagnosing IA include galactomannan (GM) and β-1,3-D-glucan (BDG). GM relatively specific to *Aspergillus*, while BDG is a broad-spectrum fungal marker with lower specificity. GM is a polysaccharide component of the *Aspergillus* cell wall and can be detected in body fluids such as blood, BALF, and cerebrospinal fluid during infection [3]. The sensitivity and specificity of GM testing vary depending on the chosen GM index threshold, sample type, and patient population. In non-neutropenic patients, the positive rate of serum GM is relatively low (approximately 40% or less) due to the clearance of GM by neutrophils and limited antibody response in blood samples [123]. In contrast, GM testing in BALF is less affected by host immunity and has superior diagnostic performance compared to serum GM [124]. In patients with AIIRD suspected of IA, BALF-GM testing may provide greater diagnostic utility, with positive detection rates up to 70% reported in Chinese cohorts [16]. However, GM detection studies specifically targeting AIIRD patients remain scarce, limiting precise estimations of sensitivity and specificity within this group. No established diagnostic GM threshold currently exists for AIIRD patients, necessitating caution when applying general IA diagnostic criteria to this population. Recent advances in rapid diagnostic technologies, such as GM lateral flow immunoassays and electronic nose (eNose) analysis of exhaled volatile organic compounds, have shown promising diagnostic potential [125–127]. Further validation studies specific to the AIIRD population are needed.

### 4.4. Molecular diagnostic technology

With increasing emergence of antifungal resistance among *Aspergillus* species, accurate species identification and antifungal susceptibility testing have become important in clinical management. In this context, molecular diagnostic techniques are helpful in both diagnosis and therapeutic decision-making. Polymerase chain reaction (PCR) enables early and rapid detection of *Aspergillus* DNA with high sensitivity and facilitates species identification [128]. Combining PCR with conventional biomarkers, such as GM and BDG, may enhance diagnostic performance compared with single assays alone [129]. As commercial PCR assays become increasingly available and laboratory workflows more standardized, PCR is emerging as an important adjunct to conventional methods for the diagnosis of IA [7]. Metagenomic next-generation sequencing (mNGS) is a promising adjunctive tool for the diagnosis of IA, offering comprehensive detection of potential pathogens from clinical specimens. A recent study in non-neutropenic patients reported that mNGS exhibited greater diagnostic sensitivity (91.7%) for IPA, higher than those of culture and serum BDG (both 33.3%) [130]. Diagnostic performance may be further improved when mNGS is combined with BALF-GM or real-time PCR [131]. However, its performance varies across clinical settings, and most available data derive from retrospective non-AIIRD populations. Moreover, mNGS results still require careful clinical correlation to distinguish invasive disease from colonization or contamination. At present, mNGS is best regarded as a complementary diagnostic tool, particularly when mixed infection is suspected or conventional tests are inconclusive.

## 5. Therapeutic strategies

Once AIIRD-IA is highly suspected or diagnosed, effective antifungal therapy should be initiated, the original immunosuppressive regimen should be evaluated, and a balance should be sought between infection control and the management of the underlying disease.

### 5.1. Antifungal therapy

Timely, adequate and full-course antifungal therapy is the key to improving the prognosis of IA. Triazole drugs are the first choice for both treatment and prevention [132]. Voriconazole, as a preferred drug verified by global phase III clinical trials, significantly improves survival rates and reduces nephrotoxicity compared with amphotericin B [133]. The new-generation broad-spectrum triazole drug isavuconazole is not inferior to voriconazole in efficacy and has better tolerance, with fewer drug-related hepatobiliary, visual and neurological adverse reactions, and can be used as an alternative first-line drug [134]. Posaconazole and itraconazole are mostly used for second-line treatment or prevention [135,136]. Amphotericin B still has clinical value in cases of triazole drug resistance or contraindication, as well as in the treatment of mixed fungal infections [137]. Echinocandins (such as caspofungin and anidulafungin) have limited efficacy as monotherapy for IA, but can play a role in combination therapy regimens [138]. Although the randomized controlled trial conducted by Marr et al. showed no difference in survival rates between voriconazole combined with anidulafungin and voriconazole monotherapy, combination therapy may be associated with survival benefits in GM-positive patients [139]. Therefore, combination therapy is generally not recommended. However, for refractory IA or drug-resistant infections, combination therapy may be considered. For non-disseminated IPA, isolated lesions that persist despite adequate drug treatment, and IA with symptoms such as intermittent hemoptysis, surgical resection may be considered, especially in the presence of necrotic lesions [137].

Antifungal therapy for IA is typically prolonged, lasting at least 6–12 weeks, with the precise duration guided by treatment response and the trajectory of immune recovery [140]. In AIIRD patients, careful attention to drug–drug interactions is also essential. Triazole are potent inhibitors of the CYP450 enzyme system, which can significantly slow the metabolism of various immunosuppressive agents (such as JAK inhibitors, cyclosporine, tacrolimus, and MMF), thereby increasing blood drug concentrations and risks of toxicity [141–143]. Key azole–immunosuppressant interactions relevant to AIIRD are summarized in Table 2. For example, co-administration of eposide can increase the drug exposure (AUC) of tacrolimus, mycophenolate mofetil, and cyclosporine by approximately 125%, 35%, and 29%, respectively [144]. Therefore, close monitoring of drug concentrations and regular assessment of liver and kidney function should be implemented.

**Table 2.** Major drug-drug interactions between immunosuppressive therapies commonly used in AIIRD and azole antifungal agents.

Immunosuppressant	Primary Metabolic Pathways	Mechanism of Interaction	Clinical Consequences	Management Recommendations
Systemic Glucocorticoids [185]	Hepatic CYP3A4 metabolism	All azoles (especially voriconazole, itraconazole, and posaconazole) can inhibit steroid metabolism.	Potentially increasing steroid exposure and leading to adverse events such as fluid retention, hyperglycemia, and adrenal suppression.	Monitor for steroid excess, particularly after azole initiation or dose escalation; Obtain morning cortisol when clinically indicated.
Calcineurin Inhibitors (Cyclosporine, Tacrolimus) [143,186]	Hepatic CYP3A4 and CYP3A5 metabolism; P-glycoprotein transporter (P-gp) substrate	All azoles increase calcineurin inhibitor levels via CYP3A/P-gp inhibition, with itraconazole, voriconazole, and posaconazole having the most pronounced effects.	Increased tacrolimus or cyclosporine concentrations, with potential risk of nephrotoxicity, neurotoxicity, hepatotoxicity, hyperkalaemia, and hypertension.	Empirically reduce calcineurin inhibitor doses at azole initiation (e.g., tacrolimus to approximately one-third of the original dose; cyclosporine to one-half with voriconazole or three-fourths with posaconazole), with trough-level monitoring within 1–3 days and frequent reassessment until stable, including after azole discontinuation.
Mycophenolate mofetil [144]	Rapid conversion to mycophenolic acid (MPA); MPA is eliminated by hepatic glucuronidation through uridine diphosphate glucuronosyltransferase (UGT) enzymes.	Isavuconazole inhibits UGT-mediated mycophenolic acid metabolism and clearance (MPA AUC increases by ∼35% on coadministration). Other azoles have minimal effect on MPA levels.	Concomitant use of isavuconazole may heighten the immunosuppressive effects and toxicity of mycophenolate mofetil (e.g. gastrointestinal disturbances, myelosuppression, leukopenia, anemia, infection).	Monitoring for drug toxicity of mycophenolate mofetil is advised when using isavuconazole. Routine mycophenolate mofetil dose adjustment is generally unnecessary for other azoles.
Cyclophosphamide [187,188]	Prodrug requiring hepatic activation via CYP2B6 (major), CYP3A4, and CYP2C9 (minor) to active 4-hydroxy-cyclophosphamide.	Azoles inhibit cyclophosphamide bioactivation via CYP2B6 and CYP3A4 blockade.	Diminished therapeutic effect of cyclophosphamide. The interaction may lead to potential treatment failure.	Avoid concomitant use where feasible. If combination is unavoidable, monitor efficacy and toxicity closely, with subsequent dose adjustment guided by clinical response.
Methotrexate [189]	Primarily renal clearance	Azole excipients (e.g. intravenous voriconazole’s sulfobutylether-β-cyclodextrin) can also impair methotrexate excretion.	Elevated methotrexate levels and prolonged exposure, leading to increased toxicity (e.g. severe mucositis, myelosuppression, nephrotoxicity, hepatotoxicity).	Close monitoring of methotrexate levels and toxicity is required. Avoid intravenous voriconazole during high-dose methotrexate therapy. Use the oral formulation or alternative antifungal.
Tofacitinib [190]	CYP3A4 metabolism (∼ 53%) and CYP2C19 metabolism (∼ 17%)	Inhibition of tofacitinib metabolism via CYP3A4 and CYP2C19 blockade.	Increased tofacitinib levels (up to ∼2-fold or more), heightening the drug’s dose-dependent risks: cytopenia, elevated liver enzymes, blood clots, and serious infections.	Reduce the tofacitinib dose to the label-recommended lower dose (e.g. 5 mg once daily) when given with strong CYP3A4 inhibitors. Monitor complete blood count, liver function, and signs of infection at baseline and again 4–8 weeks after treatment initiation.
Upadacitinib [191]	CYP3A4 metabolism	Azoles may slow the clearance of upadacitinib by affecting hepatic or intestinal enzyme CYP3A4 metabolism.	The risks of dose-dependent side effects increase (e.g. hepatic enzyme elevations, dyslipidemia, infection). Prolonged high drug levels may potentiate JAK-inhibitor class warnings (e.g. thrombosis, cardiovascular events, immunosuppression).	If a strong CYP3A4 inhibitor is needed, use the lowest effective upadacitinib dose. Continue routine safety monitoring, including complete blood count, liver enzymes, and clinical surveillance for infection.
Baricitinib [192]	Primarily renal elimination of parent drug (≈ 75% excreted unchanged in urine); minimal metabolism (≈ 6% via CYP3A4).	No significant interaction between baricitinib and azoles because baricitinib’s CYP metabolism is minimal.	Baricitinib has a low risk of azole interactions due to its predominant renal excretion and minimal CYP metabolism.	No azole-specific dose adjustment is generally required.
Tocilizumab [193]	--	CYP450 induction via IL-6 blockade: IL-6 suppresses CYP3A4 activity via the JAK/STAT3 pathway, and tocilizumab reverses IL-6–mediated suppression of CYP3A4, and increases its activity, thereby increasing the metabolism of azoles.	Reduced azole concentrations during co-therapy and potential treatment failure.	Avoid initiating tocilizumab in patients who require stable azole exposure when feasible. If overlap is unavoidable, perform therapeutic drug monitoring of azole and adjust the azole dose to maintain target levels. Monitor closely for clinical signs of fungal relapse.

Note: Monitoring suggestions in this table are pragmatic recommendations derived from prescribing information, therapeutic drug monitoring guidance, and clinical pharmacology studies, rather than AIIRD-specific comparative trials. Monitoring intensity should be individualized according to the azole selected, organ function, and whether the interacting drug is being started, stopped, or dose-adjusted. The above table highlights only the combinations requiring dose adjustment, monitoring, or other management for patient safety. Other biologics (e.g. TNF inhibitors, rituximab, abatacept, etc.) and traditional immunosuppressants (e.g. azathioprine, hydroxychloroquine) are not known to have clinically significant interactions with azole antifungals, as they are not metabolized by cytochrome P450 pathways. All azoles mentioned (including voriconazole, itraconazole, posaconazole, and isavuconazole) inhibit CYP3A4 to varying degrees and can increase exposure to multiple immunosuppressants. Voriconazole: a potent inhibitor of CYP3A4 as well as CYP2C19 and CYP2C9; itraconazole: a potent inhibitor of CYP3A4 and P-gp; posaconazole: a potent CYP3A4 inhibitor; isavuconazole: a moderate CYP3A4/5 inhibitor, a mild CYP2B6 inducer, and a mild inhibitor of P-gp, OCT2 and UGT. Abbreviations: CYP, cytochrome P450 enzymes; IL-6, interleukin-6; JAK, Janus kinase; OCT2, organic cation transporter 2; P-gp, P-glycoprotein transporter; STAT3, signal transducer and activator of transcription 3; UGT, uridine diphosphate glucuronosyltransferase.

The development of new drugs is advancing. New antifungal drugs such as olorofim, fosmanogepix, ibrexafungerp, and opelconazole have shown good activity against *Aspergillus* and have entered the II/III phase clinical trial stage, providing potential options for drug-resistant or refractory cases [145–148]. At present, clinical experience with these agents in AIIRD remains minimal, and their role in this population will need to be established through future studies.

### 5.2. Immunomodulation and supportive therapy

A key principle in treating opportunistic infections is to restore the host’s immune capacity [55]. During severe infections, drugs used to control rheumatic diseases usually need to be reduced in dosage or temporarily discontinued to support clearance of the pathogen [149]. However, this approach requires careful multidisciplinary decision-making to balance control of the underlying AIIRD against infection risk, and the available guidance in this area is drawn primarily from broader immunocompromised populations rather than from dedicated AIIRD studies. Infectious disease experts can help assess infection severity and guide targeted antifungal therapy, while rheumatologists provide advice on the safe adjustment of immunosuppressive therapy and possible bridging strategies. For patients with AIIRD-IA, it is important to determine whether the dominant clinical problem is uncontrolled AIIRD itself or the active infection, and to prioritize the treatment of life-threatening disorders accordingly. When IA is active and the underlying AIIRD is relatively stable, a commonly adopted principle is to maintain immunosuppressive therapy at the lowest effective intensity that avoids disease aggravation [150]. For glucocorticoids, higher doses have generally been associated with poorer IA outcomes in observational studies [151]. Current clinical evidence does not define a universal dose or cumulative‑exposure threshold that reliably stratifies risk among AIIRD patients; in practice, clinicians often taper the glucocorticoids dosage to the lowest maintenance dose or even discontinue glucocorticoids during active infection [152]. This tapering approach reflects extrapolation from general infectious-disease principles rather than AIIRD-specific evidence, and the optimal strategy has not been formally established. In patients receiving long-term glucocorticoids, complete withdrawal may not be appropriate, particularly when there is concern for autoimmune rebound or adrenal insufficiency. In such cases, low-dose maintenance or stress-dose glucocorticoids may be required [153–155].

Other immunosuppressants and biologics are similarly often reduced or temporarily discontinued during active infection, with the decision guided by infection severity, rheumatic disease activity, and drug pharmacokinetics. For drugs with a long metabolic half-life such as leflunomide, cholestyramine may be considered to accelerate drug clearance [156]. Although reports of aspergillosis in patients using JAK inhibitors are limited, these drugs have black box warnings for severe infections and have been associated with other opportunistic fungal infections, so a degree of vigilance is still warranted [157].

Nevertheless, if the AIIRD is active or even presents as a rheumatic crisis, overly conservative immunosuppressive treatment may not be beneficial to the patient’s prognosis. In such situations, transitional strategies such as intravenous immunoglobulin (IVIG) may be considered as adjunctive immune modulation on the basis of antifungal therapy [158,159]. For patients presenting with rheumatic crisis, inflammatory storm or multi-organ dysfunction related to AIIRD, moderate immunosuppressive and anti-inflammatory treatment may still be necessary [160,161]. It should be acknowledged that the evidence supporting these strategies in the specific context of AIIRD-IA derives largely from case-level experience and expert opinion rather than from controlled studies.

After acute IA has been controlled, secondary antifungal prophylaxis may be considered in patients expected to resume potent immunosuppressive therapy. This strategy is informed in part by experience from transplant medicine, where persistent immunosuppression often necessitates ongoing antifungal coverage [140,162]. There is no one-size-fits-all regimen in the AIIRD population, and the treatment choice depends on the disease severity and drug interactions with the reinitiated immunosuppressants.

In terms of adjunctive immunotherapy, colony-stimulating factors (CSFs) can enhance the proliferation and activation of myeloid cells, and have been used to support host defense in patients with granulocytopenia [163,164]. For patients with severe lymphocyte deficiency, the infusion of donor-derived fungal-specific T lymphocytes (adoptive immunotherapy) has been explored in hematopoietic transplantation [165]. Experimental approaches such as chimeric antigen receptor (CAR)-T cell therapy have also been investigated, including Dectin-1-based CAR-T cells designed to recognize *Aspergillus fumigatus* and inhibit its growth in murine models [166]. Although these strategies are of interest, their clinical role in IA remains uncertain. In patients with AIIRD, these immunotherapies may carry risks of exacerbating underlying autoimmunity or provoking intense inflammation. Accordingly, such therapies should be considered with caution and are likely best restricted to carefully selected patients in specialized centres.

Overall, the coexistence of infection and autoimmune disease poses therapeutic challenges. Because most of the management principles outlined above are extrapolated from hematology and transplant medicine, and direct AIIRD-specific evidence remains limited, these recommendations should be regarded as pragmatic guidance rather than evidence-based standards. Further AIIRD-specific studies will be needed to refine these strategies and to support more confident clinical recommendations in the future.

## 6. Prognosis

If IA infection is not promptly diagnosed and treated, the condition is often severe, with a high risk of hypoxemia, multiple organ failure, and even death. Prognosis is particularly poor when immune function cannot be adequately restored and disseminated infection occurs. Real-world clinical data indicate several risk factors associated with poor outcomes, including high-dose GC therapy (e.g. prednisone ≥ 20 mg or > 0.3 mg/kg/d), recent steroid pulse therapy, use of immunosuppressants such as AZA or RTX, concurrent infections (e.g. coexisting viral pneumonia, cytomegalovirus viremia), advanced age, lymphopenia, decreased CD3^+^ CD4^+^ T cells, and coexisting rapidly progressive interstitial pneumonia [9,11,14,121,167].

Continuous monitoring of GM levels in serum or BALF is useful for assessing disease progression and therapeutic response. A reduction in GM index correlates positively with treatment efficacy and lower mortality rates [168]. CT examinations are of great significance for evaluating the condition. Although guidelines do not clearly specify optimal timing for follow-up imaging, initial scans at the onset of treatment and subsequent reassessments within 1–3 weeks are generally recommended to evaluate efficacy and prognosis [118,140,169–171]. The frequency of later CT re-examinations should be more individualized, based on the clinical progression, patient immune status, and radiological improvement [118].

## 7. Challenges and future directions

Despite advances in understanding IA in immunocompromised populations, research on AIIRD patients still faces many challenges. Firstly, diagnostic delays are still common, partly due to underestimation of IA risk and insufficient clinical awareness. Non-specific clinical and radiological manifestations often lead clinicians to misattribute symptoms to other infections.

Current diagnostic criteria by EORTC/MSGERC mainly target hematological malignancies, organ transplants, or ICU patients, with unproven applicability in AIIRD patients. Although the EORTC/MSGERC definitions remain useful for research standardization, they may be insufficiently sensitive in AIIRD, where many patients are non-neutropenic and do not fit traditional host-factor thresholds [172]. The 2020 revision already recognizes prolonged corticosteroid exposure and selected T-cell immunosuppressants, including calcineurin inhibitors and TNF-α blockers, as host factors for IA. However, in AIIRD, clinically relevant risks may also arise with sustained lower-dose glucocorticoid exposure, cumulative combination immunosuppression, or structural abnormalities that do not meet formal criteria. In the proof-of-concept study by Kurita et al. [4], an additional AIIRD “potential IPA” category, defined by clinical and imaging features together with positive mycological tests even in the absence of traditional host factors, increased sensitivity from 50.0% to 100.0%, suggesting that conventional definitions may miss clinically important cases. Even so, improved sensitivity is likely to be accompanied by some loss of specificity, particularly in patients with chronic rheumatic lung disease, where inflammatory lung lesions can closely resemble IA.

Imaging diagnosis is particularly challenging in patients with underlying rheumatic lung disease. AIIRD-related pulmonary involvement may itself present with organizing pneumonia, nodules, or cavitary change, making it difficult to distinguish infection from background structural abnormalities. In clinical practice, attention should be paid to new focal nodules, consolidation, cavitation, or rapidly progressive abnormalities superimposed on chronic disease, interpreted alongside prior imaging and mycological evaluation, including BALF when feasible.

In recent years, artificial intelligence (AI) has emerged as a potential tool to aid in the diagnosis of IA [173], particularly through CT-based radiomic and deep-learning models combined with clinical data [174]. Although recent retrospective studies have reported encouraging performance [174–177], the evidence remains preliminary, with most studies derived from selected pulmonary cohorts or curated image datasets, limited external validation, and no specific evaluation in AIIRD populations. Therefore, AI remains a developing tool, and its utility still needs to be validated across different clinical settings.

Patients with AIIRD face a heterogeneous spectrum of IA infections, differing in causative *Aspergillus* species, sites of infection, and clinical outcomes. Different *Aspergillus* species exhibit distinct virulence and resistance profiles, necessitating species identification and antifungal susceptibility testing to tailor therapy [22]. Infection can occur in multiple sites including the lung, brain, sinus, or skin, each presenting distinct diagnostic and therapeutic considerations [10]. Given that AIIRD patients may also present with systemic involvement, differential diagnosis with disseminated IA infections poses challenges. Beyond IA, aspergillosis also includes two other forms: allergic bronchopulmonary aspergillosis (ABPA) and chronic pulmonary aspergillosis (CPA) [41]. Distinguishing among these entities is also challenging due to the similarity of clinical manifestations. A pragmatic differential diagnosis integrates several key dimensions, considering the patient’s course of disease, underlying conditions (e.g. ABPA typically arises in asthma or bronchiectasis, CPA on pre‑existing cavities), different degrees of immunosuppression (e.g. IPA is favored by profound immunosuppression or high‑dose corticosteroids), laboratory indicators (e.g. IgE, *aspergillus*-specific IgG, GM), and different imaging patterns (e.g. central bronchiectasis or mucus impaction in ABPA; thick‑walled cavities in CPA; halo sign or air crescent sign in IPA) [178–180]. Dynamic transitions of different forms can occur in AIIRD patients. For instance, colonization or CPA may progress to a subacute invasive form if immunosuppression is escalated. Conversely, treated invasive lesions can evolve into residual cavities that may later harbor an aspergilloma [179,181]. Periodic reassessment to ensure accurate diagnosis and appropriate management is necessary.

Managing immunosuppressive therapy in AIIRD patients diagnosed with IA remains challenging due to the delicate balance between controlling disease activity and reducing infection risk. Ceasing immunosuppression risks disease flare, whereas continuing treatment may exacerbate the infection. Currently, no clinical guidelines exist to inform decisions regarding the timing and method of resuming immunosuppression after IA infection. In addition, there is no consensus on prophylactic antifungal use in rheumatology practice, and the risk-benefit ratio of prophylaxis in the AIIRD population requires assessment. Current studies predominantly consist of retrospective analyses or small sample observation cohorts, thus, large-scale studies to investigate the epidemiology, clinical characteristics, and risk factors of IA infection in AIIRD patients are required to establish high-quality evidence for individualized management.

## 8. Conclusion

IA is a serious but often underrecognized infection in AIIRD patients. Immune dysregulation, immunosuppressive therapy, and comorbidities contribute to the elevated risk. Diagnosis is difficult because symptoms and imaging findings frequently mimic rheumatic disease itself. Wider use of advanced tools—such as bronchoalveolar lavage galactomannan, PCR, and next-generation sequencing—could enable earlier detection and better outcomes. Effective management requires both timely antifungal therapy and careful adjustment of immunosuppression, best achieved through close collaboration between rheumatology and infectious disease specialists. Future priorities include AIIRD-specific diagnostic criteria, novel biomarkers, improved risk prediction, and real-world data to guide individualized management. Greater awareness and proactive strategies are essential to reduce IA-related morbidity and mortality in this vulnerable population.

## Supplementary Material

Supplemental Material

Supplementary Figure S1.jpg

## Data Availability

Data sharing is not applicable to this article as no original data were created or analyzed in this study.

## Patient and public involvement

Patients and/or the public were not involved in the design, or conduct, or reporting, or dissemination plans of this research.
