# Supplementary material for: Invasive aspergillosis in autoimmune inflammatory rheumatic diseases: epidemiology, risk factors, diagnosis, management and challenges
Source: Ann Med. 2026 Jun 25;58(1):2685285. doi: 10.1080/07853890.2026.2685285 (PMC13307391; doi:10.1080/07853890.2026.2685285)
Supplement: Supplemental Material [file IANN_A_2685285_SM7489.doc]

**Supplementary Table S1** Summary of the principal studies informing this narrative review of invasive aspergillosis (IA) in autoimmune inflammatory rheumatic diseases (AIIRD).

| **Study (Ref.)** | **Country / Region** | **Study Design** | **Sample Size** | **AIIRD Population** | **Principal Findings Relevant to IA in AIIRD** |
| --- | --- | --- | --- | --- | --- |
| Su et al., 2021 (12) | Taiwan | Nationwide population-based cohort | 24,541 SLE patients  (matched controls); ;  445 IFI episodes;  *Aspergillus spp*. accounted for 18.2% | Systemic lupus erythematosus | Provided population-level estimates of IFI risk in SLE; IFI incidence was 20.83 per 10,000 person-years and mortality for aspergillosis reached 35.7%.  Intravenous steroid exposure within 60 days was the strongest risk factor for overall IFI and major fungal subtypes. |
| Kim et al., 2009 (15) | South Korea | Multicentre retrospective study | 1155 SLE and 2004 RA patients reviewed; 12 SLE IFI cases identified, including 6 *Aspergillus spp.* infections | Systemic lupus erythematosus; Rheumatoid arthritis comparator cohort | Reported that IFI was identified in SLE but not RA in the reviewed cohort; high SLE disease activity was associated with IFI and mortality was associated with *Aspergillus* infection. |
| Silva et al., 2015 (184) | Brazil | Multicentre retrospective study | 852 childhood-onset SLE patients screened; IFI subset reported in the original study; aspergillosis included among IFI episodes | Childhood-onset systemic lupus erythematosus | Described the spectrum and clinical course of IFI in paediatric SLE and supports the point that severe fungal infections, including aspergillosis, occur in highly immunosuppressed or severe childhood-onset SLE. |
| Galmiche et al., 2023 (9) | France | Multicentre registry-based case series (RESSIF network) | 549 patients with autoimmune diseases and IFD; IA: n=84 (RA n=15 and AAV n=10 were the most frequent autoimmune diseases) | Multiple AIIRDs (RA most frequent; 15/84, 18%) | IA is a major IFD category in autoimmune diseases and that RA and AAV were the most frequent autoimmune conditions among IA cases; high-dose glucocorticoids were associated with 30-day mortality across IFDs. |
| Hennessee et al., 2025 (59) | United States | Large administrative database analysis | 20,249 RA patients with TNF inhibitors; IFI incidence 2.8 per 1,000 person-years in RA; <0.5% of RA patients developed IFI within 1 year | Rheumatoid arthritis (RA) and inflammatory bowel disease (IBD) | Estimated IFI incidence after TNF-alpha inhibitor initiation. Histoplasmosis, unspecified mycoses and candidiasis were the most common IFIs in RA; the study is useful for TNF-inhibitor IFI context but should not be presented as direct RA-IA evidence. |
| Kurita et al., 2025 (4) | Japan | Retrospective observational proof-of-concept study | 364 AIIRD patients evaluated for suspected IPA using serum galactomannan; 24 proven/probable IPA, 29 potential IPA and 311 non-IPA | Multiple AIIRDs | Proposed a “potential IPA” category based on partial fulfilment of EORTC/MSGERC components to improve diagnostic sensitivity in AIIRD patients. Both potential IPA and proven/probable IPA were associated with significantly lower survival than non-IPA and independently predicted mortality. The proposed criteria improved diagnostic sensitivity compared with the conventional EORTC/MSGERC definition. |
| Salmon-Ceron et al., 2011 (63) | France | Prospective registry (RATIO, 3 years) | 45 non-tuberculosis opportunistic infections reported in patients receiving anti-TNF therapy, including 10 invasive fungal infections | Inflammatory diseases treated with anti-TNF agents, including rheumatic diseases | Characterised drug-specific opportunistic infection patterns during anti-TNF therapy and supports clinical vigilance for severe fungal infections, including aspergillosis, particularly with monoclonal anti-TNF agents. |
| Xiong et al., 2023 (57) | International | Systematic review and meta-analysis | 26 observational studies included | Connective tissue diseases (CTD) | Identified pooled risk factors for invasive fungal infections in CTD, including lymphopenia, glucocorticoid use, and pulmonary involvement. |
| Barbosa et al., 2025 (64) | International | Systematic review of published fungal infections associated with TNF inhibitors | 65 articles; 697 fungal infection cases associated with TNF inhibitors; 517 invasive fungal infections reported | Patients receiving TNF inhibitors for inflammatory diseases, including RA and ankylosing spondylitis | Synthesised reported cases of fungal infections, including aspergillosis, associated with TNF-α inhibitor exposure; useful as background biologic-safety evidence, but the evidence is largely derived from published cases rather than denominator-based AIIRD cohorts. |
| Suwatanapongched et al., 2021 (5) | Thailand | Single-centre retrospective study | 14 hospitalised SLE patients with IPA (4 proven, 10 probable); 5 IPA alone and 9 with co-infection | Systemic lupus erythematosus | Characterised clinical and CT manifestations of IPA in hospitalised SLE patients, highlighting nodules/masses, consolidation, ground-glass opacity and angio-invasive CT features such as halo sign. |
| Chen et al., 2012 (8) | China | Single-centre retrospective study | 18 hospitalised SLE patients with IFI | Systemic lupus erythematosus | Described clinical characteristics and outcomes of IFI in Chinese SLE patients and compared risk factors against SLE patients with other infections or active disease. |
| Kunawathanakul et al., 2019 (103) | Thailand | Single-centre retrospective age- and sex-matched case-control study; (conference abstract) | 1585 adult SLE patients screened; 22 IA cases and 66 matched controls | Systemic lupus erythematosus | Compared with matched controls, SLE patients with IA had a lower median absolute lymphocyte count. History of steroid treatment was associated with IA. Low lymphocyte count, renal impairment, and steroid exposure were associated with IA in SLE. |
| Hung et al., 2018 (121) | Taiwan | Single-centre retrospective study | 6,714 SLE patients; 21 SLE patients with IA (4 proven, 17 probable); 14 deaths | Systemic lupus erythematosus | Detailed clinical characteristics and mortality predictors of IA in SLE; mortality was high and risk factors included high daily steroid dose, recent pulse steroids, immunosuppressants and CMV viraemia. |
| Lao et al., 2019 (183) | Southern China | Single-centre retrospective study | 45 SLE patients with IFD; incidence 1.1%; mould infections n=23; *Aspergillus spp.* 44.4% of all IFD casese | Systemic lupus erythematosus | Characterised the clinical features and outcomes of invasive fungal disease, including IA, in SLE. |
| Chen et al., 2024 (11) | China | Single-centre retrospective study | 415 anti-MDA5+ DM patients; 28 IPA cases; comparator IPA-negative subgroup n=98 among those with lower respiratory tract specimens | Anti-MDA5 antibody-positive dermatomyositis | Reported IPA prevalence of 6.7% and mortality of 25% in anti-MDA5+ DM. Elevated BALF galactomannan was independently associated with IPA; deaths were associated with rapidly progressive ILD, lower lymphocyte counts and *Pneumocystis jirovecii* co-infection. |
| Su et al., 2009 (14) | China | Single-centre retrospective study | 157 AAV patients; 7 developed IPA | ANCA-associated vasculitis (AAV) | Reported IPA prevalence of approximately 4.5% and mortality of 57.1% in AAV; long-term glucocorticoids, cyclophosphamide exposure and underlying lung lesions/cavities were key clinical considerations. |
| Lao et al., 2019 (16) | Southern China | Single-centre retrospective study | 6911 CTD patients; 32 CTD with invasive mycoses; *Aspergillus spp.* accounted for 81.3% | Connective tissue diseases | Reported clinical features and associated factors of invasive mycoses (including IA) in CTD; prevalence approximately 0.38%, mortality 26.9%; supported lymphopenia, glucocorticoids, pulmonary involvement and multi-agent immunosuppression as relevant susceptibility factors. |
| Shi et al., 2025 (167) | China | Single-centre retrospective study | 152 CTD-ILD patients; 54 with IPA and 98 without IPA; among the IPA group, 30 deaths and 24 survivors | CTD-associated interstitial lung disease | CTD-ILD patients with IPA had lower CD3^+^CD4^+^ T-cell counts than CTD-ILD controls, and that lower CD3^+^CD4^+^ T-cell counts were associated with severity and poorer survival. |
| Baliga et al., 2024 (13) | India | Case series and literature review | Small case series with literature review | ANCA-associated vasculitis | Illustrated the diagnostic and therapeutic dilemma of managing invasive fungal infection, including IA, in AAV, where infection may mimic or complicate vasculitis activity. |
| Katz et al., 1996 (182) | Israel | Case series and literature review | 3 newly reported SLE patients with invasive aspergillosis; 23 previously published English-language cases reviewed | Systemic lupus erythematosus | Provided an early description of aspergillosis in SLE and helped establish IA as a rare but severe opportunistic complication in lupus. |
| Shadrach et al., 2020 (18) | India | Case report | 1 patient | Systemic sclerosis (SSc-ILD) | Reported subacute invasive pulmonary aspergillosis coexisting with SSc-ILD, supporting chronic structural lung disease plus immunosuppression as a plausible risk context. |
| Attaway et al., 2016 (19) | United States | Case report | 1 patient | Ankylosing spondylitis | Described subacute invasive pulmonary aspergillosis arising in the setting of ankylosing spondylitis. |
| Kennedy et al., 2005 (20) | United States | Case report | 1 patient | Ankylosing spondylitis on infliximab | Reported pulmonary aspergillosis following infliximab therapy for ankylosing spondylitis. |
| Pamuk et al., 2005 (88) | Turkey | Case report | 1 patient | Ankylosing spondylitis | Described chronic necrotising pulmonary aspergillosis as a presenting manifestation in ankylosing spondylitis with structural lung involvement. |
| Şeyhoğlu et al., 2018 (61) | Turkey | Case report with literature review | 1 patient + reviewed cases | Adult-onset Still's disease | Reported pulmonary aspergillosis after infliximab therapy and reviewed similar cases, adding case-based support for biologic-associated IA vigilance. |
| Robinett et al., 2013 (117) | United States | Case report | 1 patient | Antiphospholipid syndrome | Reported IA presenting as a mimic of catastrophic antiphospholipid syndrome, highlighting diagnostic overlap. |

*Notes:* Studies are grouped by approximate scale and methodological tier rather than by formal quality scoring, in keeping with the narrative scope of this review. For studies primarily reporting invasive fungal infections (IFI) rather than invasive aspergillosis (IA) alone, aspergillosis-specific data are shown where available. Mixed-population studies are retained only when they provide contextual evidence relevant to IA risk, diagnosis, or outcomes in AIIRD. Sample sizes refer to the relevant AIIRD-IA subset where this could be derived from the original publication; in some studies the precise IA-affected subgroup is not separately reported, and the total cohort size is shown instead. This table is not exhaustive of all literature cited in the review but is intended to give the reader an overview of the principal evidence underpinning the AIIRD-specific observations summarised in the text and in Table 1.

*Abbreviations:* AAV, ANCA-associated vasculitis; AIIRD, autoimmune inflammatory rheumatic diseases; ANCA, antineutrophil cytoplasmic antibody; CTD, connective tissue disease; DM, dermatomyositis; IA, invasive aspergillosis; IFI, invasive fungal infection; ILD, interstitial lung disease; IPA, invasive pulmonary aspergillosis; MDA5, melanoma differentiation-associated gene 5; RA, rheumatoid arthritis; SLE, systemic lupus erythematosus; SSc, systemic sclerosis; TNF-α, tumour necrosis factor-alpha.

**Supplementary Figure S1.** Suggested diagnostic algorithm for IA in patients with AIIRD. The five-step workflow covers clinical suspicion and host-risk assessment, imaging, microbiological and molecular workup, EORTC/MSGERC classification, initiation of targeted antifungal therapy with multidisciplinary management and modulation of immunosuppression, and serial clinical, imaging, and biomarker reassessment. The lower panel summarizes the principal AIIRD-specific caveats relevant to clinical interpretation. This algorithm is intended as a practical clinical aid only and has not been prospectively validated; clinical judgement remains essential.
