# Supplementary figures and images for: Invasive aspergillosis in autoimmune inflammatory rheumatic diseases: epidemiology, risk factors, diagnosis, management and challenges
Source: Ann Med. 2026 Jun 25;58(1):2685285. doi: 10.1080/07853890.2026.2685285 (PMC13307391; doi:10.1080/07853890.2026.2685285)

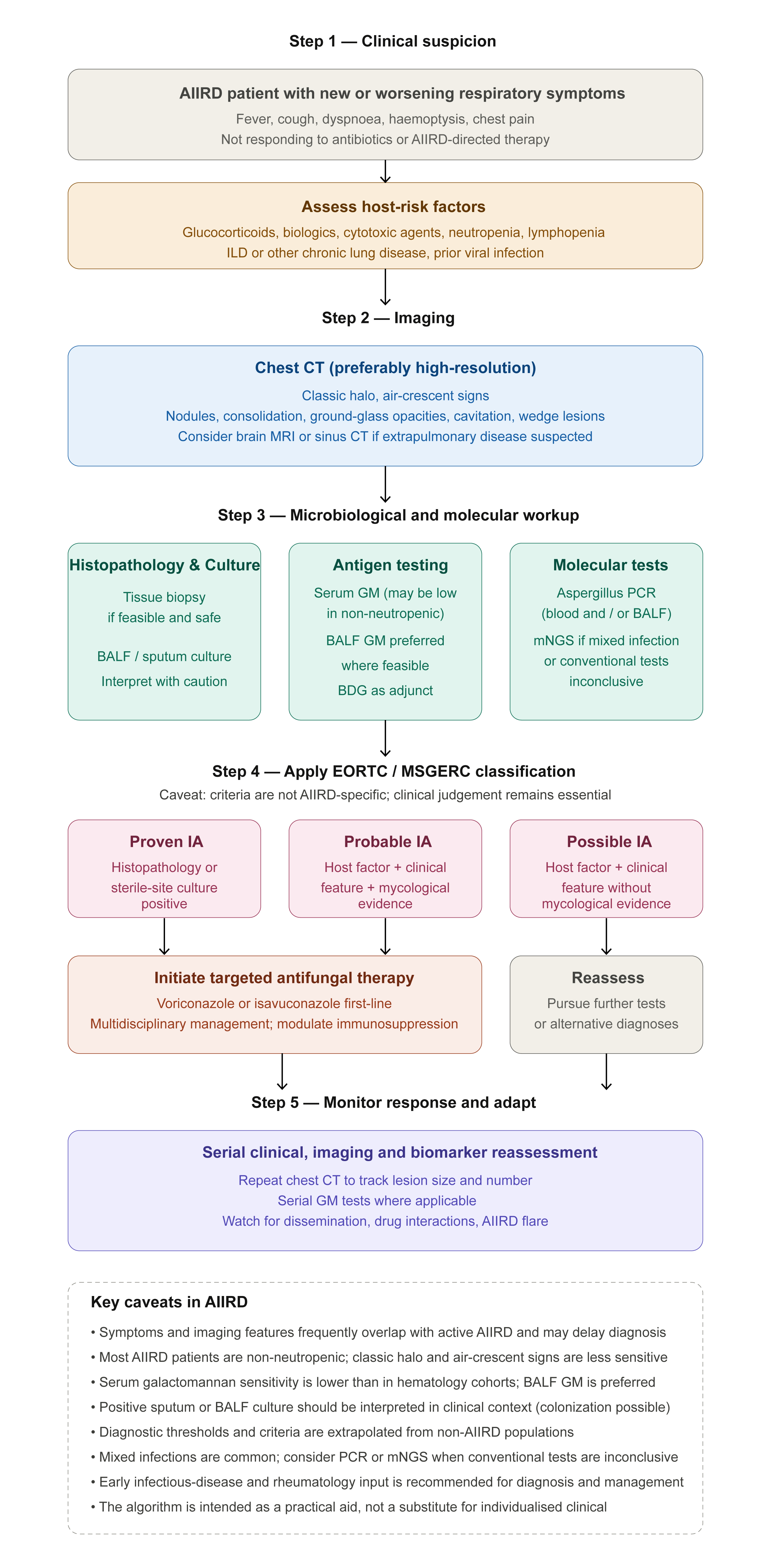

Supplement: Supplementary Figure S1.jpg [file IANN_A_2685285_SM9500.jpg]
